# Analysis of 1051 ICD Leads Extractions in Search of Factors Affecting Procedure Difficulty and Complications: Number of Coils, Tip Fixation and Position—Does It Matter?

**DOI:** 10.3390/jcm13051261

**Published:** 2024-02-23

**Authors:** Andrzej Kutarski, Wojciech Jacheć, Paweł Stefańczyk, Wojciech Brzozowski, Andrzej Głowniak, Dorota Nowosielecka

**Affiliations:** 1 Department of Cardiology, Medical University of Lublin, 20-059 Lublin, Poland; a_kutarski@yahoo.com (A.K.);; 2 2nd Department of Cardiology, Faculty of Medical Sciences in Zabrze, Medical University of Silesia, 40-055 Katowice, Poland; wjachec@interia.pl; 3 Department of Cardiology, The Pope John Paul II Province Hospital of Zamość, 22-400 Zamość, Poland; 4 Department of Cardiac Surgery, The Pope John Paul II Province Hospital of Zamość, 22-400 Zamość, Poland

**Keywords:** ICD lead extraction, dual-coil lead extraction, passive fixation lead extraction, lead extraction complexity

## Abstract

**Background**: Implantable cardioverter-defibrillator (ICD) leads are considered a risk factor for major complications (MC) during transvenous lead extraction (TLE). **Methods**: We analyzed 3878 TLE procedures (including 1051 ICD lead extractions). **Results**: In patients with ICD lead removal, implant duration was almost half as long (69.69 vs. 114.0 months; *p* < 0.001), procedure complexity (duration of dilatation of all extracted leads, use of more advanced tools or additional venous access) (15.13% vs. 20.78%; *p* < 0.001) and MC (0.67% vs. 2.62%; *p* < 0.001) were significantly lower as compared to patients with pacing lead extraction. The procedural success rate was higher in these patients (98.29% vs. 94.04%; *p* < 0.001). Extraction of two or more ICD leads or additional superior vena cava (SVC) coil significantly prolonged procedure time, increased procedure complexity and use of auxiliary or advanced tools but did not influence the rate of MC. The type of ICD lead fixation and tip position did not affect TLE complexity, complications and clinical success although passive fixation reduces the likelihood of procedural success (OR = 0.297; *p* = 0.011). Multivariable regression analysis showed that ICD lead implant duration ≥120 months (OR = 2.956; *p* < 0.001) and the number of coils in targeted ICD lead(s) (OR = 2.123; *p* = 0.003) but not passive-fixation ICD leads (1.361; *p* = 0.149) or single coil ICD leads (OR = 1.540; *p* = 0.177) were predictors of higher procedure complexity, but had no influence on MC or clinical and procedural success. ICD lead implant duration was of crucial importance, similar to the number of leads. Lead dwell time >10 years is associated with a high level of procedure difficulty and complexity but not with MC and procedure-related deaths. **Conclusions**: The main factors affecting the transvenous removal of ICD leads are implant duration and the number of targeted ICD leads. Dual coil and passive fixation ICD leads are a bit more difficult to extract whereas fixation mechanism and tip position play a much less dominant role.

## 1. Introduction

What is New? The long-term reliability of ICD leads is still a significant problem as they are more prone to malfunctions than pacing leads. Transvenous lead extraction is of key importance in ICD lead management. Several publications (presented in the Discussion) have reported that transvenous extraction of ICD leads is associated with an increased risk of developing major complications. The well-known patient-related TLE risk factors (age, gender, general health) cannot be controlled. However, the question is to what extent the decisions made by the doctor implanting an ICD system (choice of an ICD lead model, method of implantation, management of dysfunctional leads) can reduce the complexity of ICD lead extraction and its major complications. To date, only ICD lead implant duration and the presence of a second coil in the SVC have been identified as important factors, although the conclusions from studies in smaller populations are contradictory. Moreover, there are even doubts about whether the extraction of an ICD lead is more dangerous than the extraction of a pacing lead. The present study is a broad analysis of factors affecting the level of difficulty and complexity of ICD lead extraction and its complications.

More and more patients are receiving intracardiac ICD leads and their malfunction and infections result in an increase in the rate of their removal [1,2,3,4,5,6,7,8,9,10,11,12,13,14,15]. Available evidence suggests that the number of single-coil lead implantations is increasing [14,16,17] with satisfying outcomes [17,18,19]. Furthermore, the tip of the lead is more often placed in the extra-apical/septal position, and active fixation leads are becoming more and more popular [20].

Transvenous lead extraction (TLE) is a key component of lead management, including ICD lead management, and lead extraction is the optimal choice in many or even most situations [21,22,23]. Lead extraction is a highly effective procedure but carries a risk of major complications [21,22,23]. For a very long time, the necessity to extract an ICD lead has been considered a risk factor for developing major complications [2,9,10,11,13,14] although a few reports have not confirmed this observation [8,12,15]. In the last twenty years, ICD leads have become thinner and more isodiametric; however, they can still contribute to major complications [10,14,24], and in studies addressing lead extraction complexity ICD leads were regarded as a risk factor for more difficult and complicated procedures requiring the use of advanced techniques and tools [7,8,12,14,15,25,26,27]. In addition, the mechanism of lead fixation seems to be important as the removal of a passive fixation lead increases procedure complexity [25,28]. Because of possible interference, the decision to replace a malfunctioning ICD lead or to abandon it and implant a new one raises even more controversy than in pacing lead management [29]. When considering our ability to minimize the risk of major complications during ICD lead replacement, we should consider our ability to influence all risk factors for major lead extraction complications. We have no influence on some of them, such as the patient’s young age and gender (if there are indications for the implantation of an ICD system), but there may be a chance of reducing the potential risk of major complications and procedure complexity when deciding about the lead type (the number of coils and fixation mechanism), the position of the lead tip during implantation and the number of ICD leads to be extracted per patient. This study therefore set out to gain a deeper understanding of the relationship between physician-dependent factors and the course of the procedure taking advantage of our computerized database of 3878 lead extractions.

## 2. Goal of the Study

The general aim of the study was to examine the influence of implanting physician decisions on the complexity of ICD lead extraction and the risk of major complications. The specific aim of the study was to investigate whether the type of ICD leads (the number of coils, fixation mechanism) increases procedure difficulty and complexity and the risk of developing major complications. Additional goals were to determine whether removal of ICD leads was per se more difficult than extraction of pacing leads, and to examine the impact of the number of ICD leads to be extracted and ICD lead implant duration on the level of procedure difficulty and the risk of major complications.

## 3. Methods

### 3.1. Study Population

All 3878 transvenous lead extraction (TLE) procedures performed between March 2006 and April 2023 at a single high-volume center were reviewed. Patient clinical characteristics, cardiac implantable electronic device (CIED) system and history of pacing, data on targeted leads, TLE complexity, efficacy and outcomes were retrospectively analyzed. The study population consisted of 3804 patients of whom 1051 had one or more ICD leads removed whereas 2753 patients underwent pacing lead extraction (control group). The 74 ICD and CRT-D patients with only pacing lead replacement and retained active ICD leads were excluded from further analysis. In the study group of 1051 patients with an ICD lead, the mean age was 62.73 years, 18.84% were women and 35.59% of procedures were performed for infectious reasons. In the control group of 2753 individuals with a pacemaker, the mean age was 67.34 years, 45.80% were women and 30.48% of procedures were performed for infectious reasons.

### 3.2. Lead Extraction Procedure

#### 3.2.1. Definitions

Indications for TLE, procedure effectiveness and complications were defined according to the recent TLE recommendations (2009 and 2017 Heart Rhythm Society (HRS) consensus and 2018 European Heart Rhythm Association (EHRA) guidelines) [21,22,23]. The efficacy of TLE was expressed as the rate of procedural success which was defined as the removal of all targeted leads and lead material from the vascular space with the absence of any permanently disabling complication or procedure-related death. Clinical success which was defined as the removal of targeted lead material from the vascular space or retention of a small portion of the lead (4 cm) that does not negatively impact the outcome goals of the procedure [21,22].

The complications of TLE were also defined as major complications being those that were life-threatening, resulted in significant or permanent disability or death or required surgical intervention [21,22,23].

The risk of major complications (MC) related to TLE (points, percentage) was assessed using the SAFeTY TLE score, an online tool available at http://alamay2.linuxpl.info/kalkulator/ (accessed on 28 January 2020) or https://usuwanieelektrod.pl/ [30]. The ELECTRa Registry Outcome Score (EROS) was used for the prediction of significant procedural complications that required emergent surgical intervention (1–3 scale) [24]. Evaluation of procedure complexity was based on the MB score showing the need for the use of advanced tools to achieve TLE success (0–6 points) [25], LED index referring to lead extraction difficulty based on fluoroscopy times (0–50 points) [26], and Advanced TLE Techniques (Mazzone) score to predict the necessity of using advanced extraction techniques (0–4 points) [27]. 

Because each of the three commonly used procedure complexity indicators assesses one of three aspects of the issue we have elaborated although already published, comprehensive procedure complexity indicator that simultaneously represents all three forms of procedure difficulty (time and labor intensity of the procedure, the need to use advanced tools and advanced techniques) [28]. The fourth (new) complexity marker was the Complex Indicator of the Difficulty of the TLE (CID-TLE) which includes global sheath-to-sheath time for extraction of all leads >20 min (2 points), average duration of single lead extraction (sheath-to-sheath time) >12 min (2 points) and the necessity of using metal sheaths or Evolution/TightRail, alternative approach or lasso catheters or basket catheters (one point each). The sum of points was the value of CID-TLE. The procedure was deemed to be difficult when CID-TLE was ≥2 [28].

#### 3.2.2. Technical Problems during TLE

Unexpected procedure difficulties so-called technical problems during TLE, i.e., the situations that increased procedure complexity but were not complications [31]. They included break of extracted leads, loss of broken lead fragments when the main part of the lead was dissected and removed but both free ends remained in place, mobile lead fragments which flowed usually into the pulmonary vascular bed [31], blockage in lead venous entry/subclavian region preventing entry into the subclavian vein with a polypropylene catheter, Byrd dilator collapse/fracture [31,32], lead-to-lead adhesion [31], use of alternative approach [31] and dislodgement of functional leads [31].

### 3.3. Dataset and Statistical Methods

#### 3.3.1. Creation of the Subgroups for Analysis of Events and Patients

**Figure 1:**
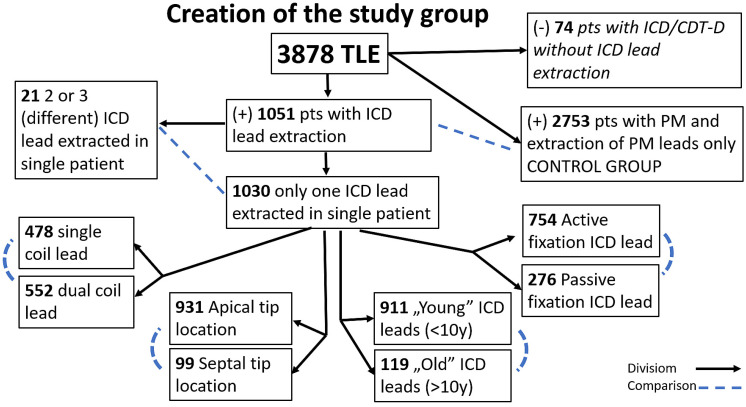
Creation of the study groups and subgroups and planned comparisons.

The entire group of 3808 TLE procedures was divided into several groups and subgroups (Figure 1). The 1051 patients with removed ICD leads were subdivided according to the purpose of analysis as depicted in Figure 1. Patients with two or more ICD leads to be removed were excluded from further analysis because of different construction, tip position and implant duration. Demographic data and basic clinical characteristics in the subgroups are summarized in tables. We are aware of the fact that the presence of abandoned leads in all subgroups affects the final result; however, a “photo” represents the “real world”. Excluding the patients with abandoned leads from analysis would reduce the material by 1/3 and show a non-existent “ideal world”.

#### 3.3.2. Procedure Information

A standard step-wise approach was used in all patients, as previously described [15]. Lead dilatation was initiated using mechanical systems (i.e., polypropylene Byrd dilator sheaths; Cook^®^ Medical, Leechburg, PA, USA), primarily via the subclavian vein on the side of the implanted device. We used alternative vascular access and/or additional tools only if technical difficulties arose (e.g., Evolution [Cook^®^ Medical], TightRail [Spectra-netix/Phillips, Colorado Springs, CO, USA], lassos, basket catheters). Laser and electrosurgical dissection sheets were not used.

#### 3.3.3. Statistical Methods 

All continuous variables are presented as the mean ± standard deviation. The categorical variables are presented as numbers and percentages. The significance of differences between the groups was determined using the nonparametric Chi2 test with Yates correction or the unpaired Mann–Whitney U test, as appropriate. The Bonferroni correction was applied, considering the value of *p* < 0.0125 as statistically significant. Survival after TLE was compared using the log rank test. To determine the impact of extracted leads on TLE complexity, clinical success, procedural success and presence of major complications univariable and multivariable regression analysis was used. The variables achieving *p* < 0.1 under the univariable regression model were entered into a multivariable model. For regression analysis, a *p*-value less than <0.05 was considered statistically significant. Statistical analysis was performed with Statistica 13.3 (TIBCO Software Inc., Tulsa, OK, USA). 

#### 3.3.4. Approval of the Bioethics Committee

All patients gave their informed written consent to undergo TLE and use anonymous data from their medical records, approved by the Bioethics Committee at the Regional Chamber of Physicians in Lublin no. 288/2018/KB/VII (the date of issue 27 November 2018). The study was carried out in accordance with the ethical standards of the 1964 Declaration of Helsinki.

## 4. Results

First, we analyzed the influence of the number of coils on the course of the extraction procedure. Table 1 summarizes the results of comparison between the subgroups.

Patients with dual-coil ICD leads that were slightly younger at the time of system implantation had more system-related procedures and, above all, longer implant duration (73.68 months vs. 64.83 months). The scores of procedure risk and complexity indicated a comparable risk of major complications (EROS score) but the MB score, LED index, ALET score and LECOM score suggested a higher level of procedure complexity compared to patients with single coil leads (16.37% vs. 14.67%). 

Patients with two or more ICD leads removed during one procedure were significantly different from those undergoing extraction of a single-coil lead. They were younger at first CIED implantation, had higher EF, almost twice as long implant duration, multiple leads in the heart and more CIED-related procedures before lead extraction. Although the SAFeTY score suggested a higher risk of major complications (2.51% vs. 0.84%), no major complications occurred in this group. Most of the prognostic scales (MB, LED, ALET) and LECOM (42.99% vs. 14.67%) correctly indicated a higher probability of a complex procedure compared to patients with a single-coil lead. 

Despite fewer extracted leads per patient but longer implant duration (69.71 months vs. 57.24 months), the level of difficulty and complexity of double-coil lead extraction (apart from longer single lead extraction time—8.59 min vs. 6.94 min) and the rate of major complications did not differ significantly from those in patients undergoing extraction of a single-coil lead. When two or more ICD leads were removed in one patient, the level of extraction difficulty and complexity was significantly higher, but there were no major complications. Table 2 shows that when using mechanical systems (we cannot say anything about the efficiency of laser systems) extraction of dual-coil leads is slightly longer and therefore more laborious.

Table 3 compares 21 patients undergoing extraction of two or more ICD leads with 1030 patients in whom only one ICD lead was removed. It is clear from the table that procedure duration, unexpected procedure difficulties, procedure complexity (based on the CID-TLE score—66.67% vs. 14.08%) and need to use auxiliary or advanced tools were significantly higher in patients undergoing removal of multiple ICD leads (38.10% vs. 7.97%). More interestingly, the mortality rate in these patients was not higher despite longer FU periods (younger patients in better general health).

A comparison of pacing and ICD lead extraction procedures revealed several differences. In patients with ICD leads, TLE was more often performed due to infective endocarditis with or without pocket infection (25.88% vs. 21.03%) and mechanical lead damage (electrical failure) (34.63% vs. 23.90%). Moreover, implant duration was almost half as long (69.69 months vs. 114.0 months), procedure duration, procedure-related unexpected difficulties, procedure complexity (based on the CID-TLE score—15.13% vs. 20.78%) and need to use auxiliary or advanced tools was significantly lower in patients undergoing ICD lead removal (8.57% vs. 15.77%). In addition, major complications (2.62% vs. 0.67%) and partial radiographic success (remained tip or < 4 cm lead fragment) were significantly more frequent (5.01% vs. 1.32%) in patients undergoing removal of pacing leads (in pacemaker patients). As a result, the rate of procedural success was lower in these patients (94.04% vs. 98.29%).

However, these findings do not indicate that extraction of ICD leads is easier, simpler, less laborious and less risky because the groups are incomparable in terms of implant duration. Nevertheless, it can be said with certainty that ICD leads are removed sooner after their implantation (for infectious and non-infectious reasons), which has a significant impact on the lead body dwell time (69.69% vs. 114.0 months) and the resulting implant duration derivatives: the difficulty of the procedure, complexity and safety of extraction.

More active than passive fixation ICD leads were extracted (73.20%). The dwell time of the active fixation ICD leads was shorter (59.02 months vs. 77.32 months) and as a result, procedure duration was shorter, procedural success was more frequent (98.94% vs. 96.38%) and partial radiographic success was less likely (0.80% vs. 2.90%). The extraction of passive fixation ICD leads was a bit more complex but the differences turned out to be statistically insignificant (Table 4). 

The removed ICD leads most often had the tip positioned in the apex of the right ventricle (90.04%) and there were no significant differences in procedure duration and complexity, rates of major complications, partial radiographic success and ultimately procedural success between extraction of leads with their tips in the apical and extra-apical/septal position.

In this study, the mean ICD lead dwell time was 68.80 months, with a predominance of leads with implant duration less than 10 years (88.45%). Removal of ICD leads at >10 years post-implant was associated with a longer duration of all-lead dilatation (18.40 min vs. 11.84 min) and average time of single lead removal (12.07 min vs. 7.27 min), higher incidence of unexpected procedure difficulties (technical problems), higher CID-TLE scores (0.78 vs. 0.33), near five times more frequent need to switch to mechanical rotational sheaths (Evolution or TightRail), more frequent partial radiographic success and less frequent procedural success (96.64% vs. 98.46%) (Table 4). These findings show that the dwell time of ICD leads has a significant influence on the level of TLE difficulty and complexity, and ultimately, on procedural success. In this study, longer lead dwell time was not associated with a significantly higher incidence of major complications and procedure-related death.

Univariable regression analysis showed that the variables were not associated with major complications and clinical success. Extraction of passive fixation ICD leads to a decrease (OR = 0.297; *p* = 0.011) probability of procedural success. The age of extracted ICD leads, passive fixation and the number of coils affected procedure complexity (Table 5).

Multivariable regression analysis showed that ICD lead dwell time ≥120 months and the number of coils but not passive fixation and single coil ICD leads were the most powerful predictors of higher procedural complexity: ICD lead dwell time ≥120 months; OR = 2.956 [95% CI (1.897–4.606); *p* < 001], number of coils; OR = 2.123 [95% CI (1.285–3.509); *p* = 0.003], passive fixation ICD lead; OR = 1.361 [95% CI (0.898–2.064); *p* = 0.149] and single coil ICD lead; OR = 1.540 [95% CI (0.822–2.887); *p* = 0.177]. 

## 5. Discussion

This study revealed that in patients undergoing ICD lead extraction implant duration was almost half as long (69.69 months vs. 114.0 months) compared to pacemaker patients, whereas levels of procedure difficulty and complexity, and rates of major complications (0.48% vs. 1.27%) and partial radiographic success (1.32% vs. 5.01%), were significantly lower. As a result, the procedural success rate was higher in ICD patients (98.29% vs. 94.04%). In this regard, these results corroborate the findings of other studies [8,12,15,28]. However, these observations do not justify the statement that extraction of ICD leads is easier, simpler and less risky because the groups are incomparable in terms of implant duration. Nevertheless, it is obvious that implant duration is the most important factor that affects ICD lead extraction and its derivatives: procedure difficulty, complexity and safety. To confirm the view that ICD lead extraction is a risk factor for major TLE complications [9,10,11,13,14,24], the influence of targeted lead dwell time should be eliminated and a completely different study should be designed in which patients with ICD and pacing lead removal would be matched by implant duration, age and gender. However, this issue was not the main goal of the present study.

Another controversial topic is the influence of an additional (proximal) coil on the level of difficulty and complexity of ICD lead extraction. Previous studies observed inconsistent results with several reports suggesting that this lead model is a risk factor for major complications [2,14], in contrast to other reports [3,7].

Many investigators are of the opinion that the presence of an additional (proximal) coil adversely affects procedural difficulty and complexity [2,7,14,26]. This study demonstrated that additional (SVC) coils only prolong the procedure when using mechanical systems and do not influence the development of major complications (such as SVC laceration), but to be fair, there is one report indicating that this additional coil does not affect the course of the procedure [3].

We demonstrated also that extraction of two or more ICD leads in the same patient prolongs the procedure, making it more difficult and more complex but does not affect the occurrence of major complications. In reviewing the literature, no data were found on the final result of removing multiple ICD leads. But a remarkably increased level of difficulty and complexity of the procedures where abandoned ICD leads have an extremely long implant duration unveils the dark side of ICD lead abandonment.

We were unable to prove the influence of the ICD lead fixation mechanism on the level of extraction complexity and complications. There were more unexpected technical problems; the removal of passive fixation ICD leads was a bit more complex but the differences turned out to be statistically insignificant. As is well known, extracting non-isodiametric passive fixation leads is more difficult compared to the isodiametric ones; however, we have not encountered any reports on this topic, although removal of passive fixation leads and ICD leads increases the likelihood of using advanced tools in lead extraction procedures [25].

Active fixation leads allow positioning in the extra-apical pacing sites, and septal positions seem to be more favorable, especially in CRT-D systems [20]. Unfortunately, there are no studies on how ICD lead tip position translates into ease of extraction. In the present study, we did not find any influence of ICD lead tip position (apical vs. septal) on extraction complexity, complications and outcomes. We know from experience that dissection of the distal coil in RVA may be laborious but it was not confirmed in statistical analysis.

The dwell time of targeted ICD leads seems to be of crucial importance [4,5,11,13,15,28]. It is considered a risk factor for both major complications [4,11,13] and increased difficulty and complexity of the procedure [5,15,28]. We showed that implant duration over 10 years is associated with longer lead dissection, higher incidence of unexpected procedure difficulties, five times more frequent need to use mechanical rotational sheaths, more frequent partial radiographic success and less frequent procedural success. Dwell time of ICD leads is an important factor that increases the level of procedure difficulty and complexity, and ultimately the rate of procedural success but it is not associated with a significantly higher incidence of major complications and procedure-related deaths. A recently published analysis of the extraction results of 885 ICD leads (average implant duration 8 years) performed by Hayashi K et al. showed, that the TLE outcome expressed as procedural success rate is related to the manufacturer, model and the age of the ICD leads. Linox family lead and >10 years lead age were independent predictors of incomplete lead removal and ICD lead age >15 years and longer lead extraction times are predictors of major complications [33]. The GALLERY Register also shows that 10 years is the safety limit for ICD lead extraction [34]. Furthermore, our studies confirmed the known fact that the long-term reliability of ICD leads is limited by an earlier occurrence of failure than is the case of pacemaker leads [35,36,37]. In our study, we analyzed slightly different factors that may influence the ICD lead extraction outcome.

## 6. Conclusions

The main factor affecting the course of ICD lead extraction is implant duration and the number of targeted ICD leads.

Dual coil and passive fixation ICD leads are a bit more difficult to extract whereas fixation mechanism and lead tip position play a much lesser role.

Extraction of ICD leads is associated with lower rates of major complications compared to extraction of pacing leads.

## 7. Study Limitations

There are some limitations in this study. It presents a retrospective analysis of prospectively collected data. All procedures were performed using all types of mechanical systems but not laser-powered sheaths. The study aimed to assess the effectiveness and outcomes of extracting different models of ICD leads. But procedure complexity and major complications are related to the entire procedure and not to the extraction of one type of the lead(s) because removal of ICD leads was often accompanied by extraction of other pacing leads and we can never be sure which of these leads caused the complication. Patients with ICD leads often had abandoned leads or newer leads implanted during system upgrade procedures. Also, procedure difficulties were related not only to the removal of a specific (ICD) lead but also to the presence of additional leads. Last but not least, this is the presentation of a single, very experienced center. Therefore, the outcomes of the extraction procedures may not represent the overall safety and efficacy of transvenous extraction of leads especially with long implant duration.

## Figures and Tables

**Table 1:** Single vs. dual coil lead extraction. Characteristics of the subgroups (demographic, clinical, risk factors for increased procedure complexity and complications).

Patient-Related Risk Factors for Procedure Complexity, Major Complications and Indications for TLE	Group 1 Extraction of One Single Coil ICD Lead	Group 2 Extraction of One Dual Coil ICD Lead	Group 3 Extraction of Two ICD Leads in Single Patients
Number of patients	N = 478 Mean ± SD *n* (%)	N = 552 Mean ± SD *n* (%)	N = 21 Mean ± SD *n* (%)
		*p*: 2 vs. 1	*p*: 3 vs. 1
Clinical data			
Patient age during TLE [years]	63.24 ± 12.95	62.37 ± 13.46 *p* = 0.295	58.62 ± 18.03 *p* = 0.117
Patient age at first system implantation [years]	57.99 ± 13.35	56.28 ± 13.98 *p* = 0.045	49.19 ± 19.00 *p* = 0.003
Female	93 (19.46)	97 (17.57) *p* = 0.486	8 (38.10) *p* = 0.071
Ischaemic heart disease	272 (56.90)	324 (58.70) *p* = 0.605	8 (38.10) *p* = 0.140
NYHA functional class III and IV	145 (30.34)	128 (23.19) *p* = 0.012	2 (9.52) *p* = 0.071
Left ventricular ejection fraction [%]	38.45 ± 15.26	38.20 ± 15.02 *p* = 0.793	50.19 ± 16.31 *p* < 0.001
Charlson comorbidity index [points]	5.36 ± 3.97	5.09 ± 3.80 *p* = 0.204	4.33 ± 4.55 *p* = 0.238
Main indications for TLE (primary/predominant)			
Infective endocarditis with or without pocket infection	136 (28.45)	129 (23.37) *p* = 0.074	7 (33.33) *p* = 0.812
Local (isolated) pocket infection	47 (9.83)	55 (9.96) *p* = 0.973	0 (0.00) *p* = 0.259
Mechanical lead damage (electrical failure)	141 (29.50)	216 (39.13) *p* = 0.002	7 (33.33) *p* = 0.759
Lead malfunction (exit/entry block, dislodgement, perforation, extracardiac pacing)	103 (21.55)	112 (20.29) *p* = 0.676	4 (19.05) *p* = 0.999
Change of pacing mode/upgrading, downgrading	16 (3.35)	7 (1.27) *p* = 0.041	0 (0.00) *p* = 0.826
Other non-infectious indications *	35 (7.32)	33 (5.98) *p* = 0.459	3 (14.49) *p* = 0.449
System-related risk factors			
Longest lead dwell time before TLE [months]	64.83 ± 48.84	73.68 ± 47.81 *p* = 0.003	113.2 ± 57.33 *p* < 0.001
Global lead dwell time before TLE [years]	9.56 ± 8.63	10.75 ± 9.30 *p* = 0.034	18.83 ± 11.89 *p* < 0.001
Presence of abandoned leads before TLE	25 (5.23)	40 (7.25) *p* = 0.231	20 (95.24) *p* < 0.001
Number of leads in the heart before TLE	1.91 ± 0.88	1.81 ± 0.85 *p* = 0.064	2.62 ± 0.87 *p* < 0.001
Number of CIED procedures before lead extraction	1.65 ± 0.98	1.70 ± 0.96 *p* = 0.400	2.60 ± 0.88 *p* < 0.001
Two or more CIED procedures before TLE	209 (43.72)	282 (51.09) *p* = 0.022	21 (100.0) *p* < 0.001
Values of different scores for evaluation of risk factors for major complications or procedure complexity			
SAFeTY score (risk of MC) [points]	4.09 ± 3.35	4.00 ± 3.24 *p* = 0.651	8.02 ± 4.12 *p* < 0.001
SAFeTY score (estimated risk of MC) [%]	0.90 ± 0.02	0.84 ± 0.01 *p* < 0.001	2.51 ± 0.03 *p* < 0.001
EROS score (risk of MC) [points]	1.35 ± 0.50	1.34 ± 0.53 *p* = 0.663	1.57 ± 0.68 *p* = 0.052
2 and 3 EROS score (risk of MC)	163 (34.10)	171 (30.98) *p* = 0.317	10 (47.62) *p* = 0.299
MB score (need for advanced tools) [points]	2.71 ± 1.20	2.83 ± 1.18 *p* = 0.108	4.29 ± 0.64 *p* < 0.001
LED index (predicted procedure fluoroscopy time) [points]	6.81 ± 4.26	8.43 ± 4.19 *p* = 0.948	12.76 ± 4.46 *p* < 0.001
Advanced TLE (ALET—Mazzone—need for advanced TLE techniques [points]	2.82 ± 0.78	2.84 ± 0.78 *p* = 0.590	3.67 ± 0.58 *p* < 0.001
LECOM score (predicted procedure complexity) [points]	6.79 ± 3.72	7.34 ± 3.72 *p* = 0.018	13.04 ± 3.07 *p* < 0.001
LECOM score (predicted procedure complexity) (%)	14.67 ± 15.11	16.37 ± 15.96 *p* = 0.080	42.99 ± 18.90 *p* < 0.001

TLE—transvenous lead extraction, ICD—implantable cardioverter defibrillator, NYHA—New York Heart Association, * Other non-infectious indications: abandoned lead/prevention of abandonment (AF, redundant leads), threatening/potentially threatening lead (loops, free ends, left heart, LDTVD), other (MRI indications, cancer, painful pocket, loss of indications for pacing/ICD) and necessity of re-establishing venous access, CIED—cardiac implantable electronic devices, MC—major complications, SAFeTY-TLE, SAFeTY-TLE score [30]; MB, MB score [25]; ALET—Mazzone, Mazzone score [27]; LED, LED index [26]; EROS, ELECTRa Registry Outcome Score [24]. LECOM, Lead Extraction COMplexity score [28].

**Table 2:** Single vs. dual coil ICD lead extraction. Procedure difficulty, complexity, major complications and outcomes.

Patient-Related Risk Factors for Procedure Complexity, Major Complications and Indications for TLE	Group 1 Extraction of One Single Coil ICD Lead	Group 2 Extraction of One Dual Coil ICD Lead	Group 3 Extraction of Two ICD Leads in Single Patients
Number of patients	N = 478 Mean ± SD *n* (%)	N = 552 Mean ± SD *n* (%)	N = 21 Mean ± SD *n* (%)
		*p*: 2 vs. 1	*p*: 3 vs. 1
Procedure-related potential risk factors for major complications and increased procedure complexity			
Number of extracted leads per patient	1.69 ± 0.85	1.52 ± 0.78 *p* = 0.005	2.52 ± 0.87 *p* < 0.001
Extraction of abandoned lead(s) (any)	23 (4.81)	38 (6.88) *p* = 0.203	20 (95.24) *p* < 0.001
Extraction of passive-fixation leads (excluding LV leads)	149 (31.17)	293 (53.08) *p* < 0.001	15 (71.43) *p* < 0.001
Age of oldest extracted ICD lead [months]	57.24 ± 40.16	69.71 ± 45.16 *p* < 0.001	113.2 ± 57.33 *p* < 0.001
Longest dwell time of extracted lead per patient [months]	63.82 ± 47.98	73.12 ± 47.89 *p* < 0.001	113.2 ± 57.33 *p* < 0.001
Average dwell time of extracted lead per patient [months]	59.49 ± 41.58	70.26 ± 44.80 *p* < 0.001	88.94 ± 55.04 *p* = 0.008
TLE complexity and outcomes			
Procedure duration (sheath-to-sheath) [minutes]	11.60 ± 8.42	13.46 ± 22.05 *p* = 0.109	52.76 ± 60.01 *p* < 0.001
Average time of single lead extraction (sheath-to-sheath time/number of extracted leads) [minutes]	6.94 ± 10.67	8.59 ± 11.72 *p* = 0.019	23.62 ± 30.16 *p* < 0.001
Number of unexpected procedure difficulties per patient	1.36 ± 0.74	1.33 ± 0.75 *p* = 0.532	2.00 ± 0.89 *p* < 0.001
Two or more unexpected procedure difficulties	12 (2.51)	22 (3.99) *p* = 0.252	2 (9.52) *p* < 0.219
CID-TLE score [points]	0.34 ± 0.87	0.42 ± 0.92 *p* = 0.163	1.81 ± 1.44 *p* < 0.001
CID-TLE score ≥ 2 points	60 (12.55)	85 (15.40) *p* = 0.223	14 (66.67) *p* < 0.001
Use of additional tools			
Evolution (old and new) or TightRail	4 (0.84)	10 (1.81) *p* = 0.282	1 (4.76) *p* = 0.517
Metal sheaths and/or lasso-, basket catheters	53 (6.92)	49 (8.89) *p* = 0.280	8 (38.10) *p* = 0.008
Major complications (any)	3 (0.63)	4 (0.72) *p* = 0.848	0 (0.00) *p* = 0.281
Haemopericardium	2 (0.42)	2 (0.36) *p* = 0.740	0 (0.00) *p* = 0.142
Haemothorax	1 (0.21)	1 (0.18) *p* = 0.534	0 (0.00) *p* = 0.834
Rescue cardiac surgery	3 (0.63)	2 (0.36) *p* = 0.872	0 (0.00) *p* = 0.281
Tricuspid valve damage during TLE (severe)	0 (0.00)	1 (0.18) *p* = 0.943	0 (0.00) NA
Death, procedure-related (intra-, post-procedural)	1 (0.21)	1 (0.18) *p* = 0.534	0 (0.00) *p* = 0.834
Partial radiographic success (remained tip or <4 cm lead fragment)	4 (0.84)	10 (1.81) *p* = 0.281	0 (0.00) *p* = 0.407
Procedural success	475 (99.37)	540 (97.83) *p* = 0.071	21 (100.0) *p* = 0.281

ICD—implantable cardioverter defibrillator, N—number of patients/procedures, SD—standard deviation, TLE—transvenous lead extraction, LV (pacing) lead—a lead designed for pacing of the left ventricle, CID-TLE—Complex Indicator of the Difficulty of the TLE [28].

**Table 3:** The effect of the number of ICD leads extracted on procedure difficulty, complexity, major complications and outcome. Comparison with pacing lead extraction.

Patient-Related Risk Factors for Procedure Complexity, Major Complications and Indications for TLE	Group 3 Two or Three ICD Leads Extracted in Single Patients	Group 4 (1 + 2) Only One ICD Lead Extracted (1 + 2)	Group 5 (1 + 2 + 3) All Extraction Procedures of ICD Leads	Group 6 Only Pacing Lead(s) Present and Extracted
Number of patients	N = 21 Mean ± SD *n* (%)	N = 1030 Mean ± SD *n* (%)	N = 1051 Mean ± SD *n* (%)	N = 2753 Mean ± SD *n* (%)
		*p*: 3 vs. 4		*p*: 5 vs. 6
Main indications for TLE—(primary/predominant)				
Infective endocarditis with or without pocket infection	7 (33.33)	265 (25.73) *p* = 0.592	272 (25.88)	579 (21.03) *p* = 0.002
Local (isolated) pocket infection	0 (0.00)	102 (9.90) *p* = 0.252	102 (9.71)	260 (9.44) *p* = 0.855
Mechanical lead damage (electrical failure)	7 (33.33)	357 (34.66) *p* = 0.916	364 (34.63)	658 (23.90) *p* < 0.001
Lead dysfunction (exit/entry block, dislodgement, perforation, extracardiac pacing)	4 (19.05)	215 (20.87) *p* = 0.946	219 (20.84)	617 (22.41) *p* = 0.315
Change of pacing mode/upgrading, downgrading	0 (0.00)	23(2.23) *p* = 0.951	23 (2.19)	227 (8.25) *p* < 0.001
Other non-infectious indications	3 (14.49)	68 (6.60) *p* = 0.342	71 (6.76)	412 (11.95) *p* < 0.001
System and history of pacing				
Number of procedures before lead extraction	2.60 ± 0.88	1.68 ± 0.97 *p* < 0.001	1.70 ± 0.98	1.92 ± 1.11 *p* < 0.001
Age of oldest extracted ICD lead [months]	113.2 ± 57.33	63.93 ± 43.34 *p* < 0.001	64.92 ± 44.18	114.0 ± 80.16 *p* < 0.001
Longest dwell time of extracted lead per patient [months]	113.2 ± 57.33	68.80 ± 48.13 *p* < 0.001	69.69 ± 48.70	114.0 ± 80.10 *p* < 0.001
TLE difficulty, complexity, complications and outcomes				
Procedure duration (sheath-to-sheath) [minutes]	52.76 ± 60.01	5.00 ± 20.46 *p* < 0.001	13.40 ± 22.59	15.88 ± 23.62 *p* = 0.003
Mean time of single lead extraction (sheath-to-sheath/number of extracted leads) [minutes]	23.62 ± 30.16	7.83 ± 11.27 *p* < 0.001	8.14 ± 12.11	9.28 ± 13.02 *p* = 0.014
Number of unexpected procedure difficulties per patient	2.00 ± 0.89	1.34 ± 0.74 *p* < 0.001	1.37± 0.76	1.47 ± 0.77 *p* < 0.001
Two or more unexpected procedure difficulties	2 (9.52)	34 (3.30) *p* < 0.001	38 (3.62)	198 (7.19) *p* < 0.001
CID-TLE score [points]	5.00 ± 1.44	0.38 ± 0.90 *p* = 0.001	0.41 ± 0.93	0.62 ± 1.19 *p* < 0.001
CID-TLE score ≥ 2 points	14 (66.67)	145 (14.08) *p* < 0.001	159 (15.13)	572 (20.78) *p* < 0.001
Use of additional tools				
Evolution (old and new) or TightRail	1 (4.76)	14 (1.36) *p* = 0.710	15 (1.43)	50 (1.82) *p* = 0.492
Metal sheaths or/and lasso-basket catheters	8 (38.10)	82 (7.97) *p* < 0.001	90 (8.57)	434 (15.77) *p* < 0.001
TLE efficacy and complications				
Major complications (any)	0 (0.00)	7 (0.68) *p* = 0.329	7 (0.67)	72 (2.62) *p* < 0.001
Rescue cardiac surgery	0 (0.00)	5 (0.49) *p* = 0.200	5 (0.48)	35 (1.27) *p* = 0.049
Death, procedure-related (intra-, post-procedural)	0 (0.00)	2 (0.19) *p* = 0.840	2 (0.19)	4 (0.15) *p* = 0.755
Partial radiographic success (remained tip or <4 cm lead fragment)	0 (0.00)	14 (1.36) *p* = 0.672	14 (1.32)	138 (5.01) *p* < 0.001
Procedural success	21 (100.0)	1012 (98.25) *p* = 0.812	1033 (98.29)	2589 (94.04) *p* < 0.001
Mortality after TLE		Log rank *p* = 0.091		Log rank *p* <0.001
Survivors	14 (66.67)	559 (54.27) *p* = 0.364	573 (54.52)	1751 (63.60) *p* < 0.001
All deaths	7 (33.33)	471 (45.74) *p* = 0.364	478 (45.48)	1002 (36.40) *p* < 0.001
FU duration of patients who are still alive [years]	8.13 ± 4.14	6.39 ± 3.96 *p* = 0.045	6.42 ±3.97	6.76 ± 4.19 *p* < 0.024
FU of patients who died during the FU period [years]	3.90 ± 2.37	3.45 ± 2.83 *p* = 0.466	3.45 ±2.82	4.47 ± 3.62 *p* < 0.001

TLE—transvenous lead extraction, ICD—implantable cardioverter defibrillator, N—number of patients/procedures, SD—standard deviation, CID-TLE—Complex Indicator of the Difficulty of the TLE, FU-follow-up, observational period [28].

**Table 4:** Impact of ICD lead fixation mechanism, tip position and lead dwell time on procedure difficulty, complications and outcome.

Risk Factors in the Study Groups	Group A Passive Fixation (Single) ICD Lead Was Extracted	Group B Active Fixation (Single) ICD Lead Was Extracted	Group C RVA Tip Position of Extracted (Single) ICD Lead	Group D RVS Tip Position of Extracted (Single) ICD Lead	Group E ≥10-year-old Extracted (Single) ICD Leads	Group F <10-year-old Extracted (Single) ICD Leads
Number of patients	N = 276 Mean ± SD *n* (%)	N = 754 Mean ± SD *n* (%)	N = 931 Mean ± SD *n* (%)	N = 99 Mean ± SD *n* (%)	N = 119 Mean ± SD *n* (%)	N = 911 Mean ± SD *n* (%)
		*p*: A vs. B		*p*: C vs. D		*p*: E vs. F
System- and procedure-related risk factors						
Number of procedures before lead extraction	1.73 ± 0.91	1.66 ± 0.99 *p* = 0.326	1.68 ± 0.95	1.69 ± 1.12 *p* < 0.899	2.44 ± 0.92	1.60 ± 0.94 *p* < 0.001
Age of oldest extracted ICD lead [months]	77.32 ± 48.30	59.02 ± 40.31 *p* < 0.001	63.61 ± 43.22	66.93 ± 44.58 *p* < 0.998	150.2 ± 29.17	52.75 ± 30.36 *p* < 0.001
Longest lead dwell time per patient [months]	81.24 ± 50.96	64.25 ± 46.26 *p* < 0.001	68.37 ± 47.61	72.86 ± 52.83 *p* < 0.377	150.7 ± 33.42	58.10 ± 38.50 *p* < 0.001
TLE difficulty and complexity						
Procedure duration (sheath-to-sheath) [minutes]	14.70 ± 25.24	11.83 ± 18.36 *p* < 0.046	12.64 ± 20.65	12.22 ± 18.66 *p* < 0.88	18.40 ± 19.77	11.84 ± 20.44 *p* < 0.001
Mean time of single lead extraction (sheath-to-sheath/number of extracted leads) [minutes]	9.78 ± 14.14	7.11 ± 9.93 *p* < 0.001	7.77 ± 10.68	8.30 ± 15.80 *p* < 0.659	12.07 ± 13.45	7.27 ± 10.84 *p* < 0.001
Number of unexpected procedure difficulties per patient	1.32 ± 0.67	1.36 ± 0.78 *p* = 0.510	1.36 ± 0.76	1.13 ± 0.35 *p* = 0.001	1.27 ± 0.58	1.36 ± 0.78 *p* = 0.161
Two or more unexpected procedure difficulties	12 (4.35)	22 (2.92) *p* = 0.347	33 (3.55)	1 (1.01) *p* = 0.296	6 (5.04)	28 (3.07) *p* = 0.391
CID-TLE score [points]	0.46 ± 1.01	0.35 ± 0.85 *p* = 0.083	0.39 ± 0.91	0.31 ± 0.77 *p* = 0.428	0.78 ± 1.14	0.33 ± 0.85 *p* = 0.001
CID-TLE score ≥ 2 points	47 (17.03)	98 (13.00) *p* = 0.122	132 (14.18)	13 (13.13) *p* = 0.894	37 (31.09)	108 (11.86) *p* = 0.001
Use of additional tools						
Evolution (old and new) or TightRail	4 (1.45)	10 (1.33) *p* = 0.879	14 (1.51)	0 (0.00) *p* = 0.440	5 (4.20)	9 (0.99) *p* = 0.015
Metal sheaths or/and lasso catheters/snares, basket catheters	26 (9.42)	56 (7.44) *p* = 0.359	77 (8.28)	5 (5.05) *p* = 0.352	13 (10.92)	69 (7.58) *p* = 0.276
TLE efficacy and complications						
Major Complications (any)	2 (0.72)	5 (0.66) *p* = 0.748	7 (0.75)	0 (0.00) *p* = 0.824	1 (0.84)	6 (0.66) *p* = 0.714
Rescue cardiac surgery	1 (0.36)	4 (0.53) *p* = 0.871	5 (0.54)	0 (0.00) *p* = 0.976	1 (0.84)	4 (0.44) *p* = 0.913
Death, procedure-related (intra-, post-procedural)	1 (0.36)	1 (0.13) *p* = 0.954	2 (0.21)	0 (0.00) *p* = 0.460	0 (0.00)	2 (0.22) *p* = 0.552
Partial radiographic success (remained tip or <4 cm lead fragment)	8 (2.90)	6 (0.80) *p* = 0.023	14 (1.50)	0 (0.00) *p* = 0.440	4 (3.36)	10 (1.10) *p* = 0.113
Procedural success	266 (96.38)	746 (98.94) *p* = 0.012	913 (98.07)	99 (100.0) *p* = 0.346	115 (96.64)	897 (98.46) *p* = 0.291

TLE—transvenous lead extraction, ICD—implantable cardioverter defibrillator, N—number of patients/procedures, SD—standard deviation, CID-TLE—Complex Indicator of the Difficulty of the TLE [28].

**Table 5:** Impact of ICD lead-related parameters on complexity, major complications and success of TLE procedure, results of univariable regression analysis.

Impact of ICD Lead-Related Parameters on Complexity, Major Complications and Outcomes of TLE Procedure				
	CID-TLE ≥2 Points	Major Complications	Clinical Success	Procedural Success
Apical position of the ICD lead(s) tip	OR = 1.255 95%CI [0.673–2.337] *p* = 0.474	OR = 10.52 95%CI [0.047–2.182] *p* = 0.397	OR = 0.098 95%CI [0.001–15.26] *p* = 0.367	OR = 0.093 95%CI [0.003–3.139] *p* = 0.185
Age of extracted ICD lead(s) ≥ 120 months	OR = 3.537 95%CI [2.323–5.384] *p* < 0.001	OR = 1.234 95%CI [0.147–10.36] *p* = 0.846	OR = 0.946 95%CI [0.117–7.622] *p* = 0.958	OR = 0.465 95%CI [0.150–1.438] *p* = 0.183
Passive fixation ICD lead(s)	OR = 1.547 95%CI [1.100–2.253] *p* = 0.013	OR = 1.054 95%CI [0.204–5.441] *p* = 0.950	OR = 0.377 95%CI [0.094–1.517] *p* = 0.169	OR = 0.297 95%CI [0.116–0.760] *p* = 0.011
Single-coil ICD lead(s)	OR = 0.722 95%CI [0.510–1.022] *p* = 0.066	OR = 0.904 95%CI [0.199–4.096] *p* = 0.895	OR = 1.386 95%CI [0.329–5.835] *p* = 0.656	OR = 1.673 95%CI [0.622–4.498] *p* = 0.307
Number of coils	OR = 1.690 95%CI [1.268–2.253] *p* < 0.001	OR = 1.028 95%CI [0.290–3.646] *p* = 0.966	OR = 0.830 95%CI [0.254–2.711] *p* = 0.758	OR = 0.634 95%CI [0.301–1.338] *p* = 0.231

ICD—implantable cardioverter defibrillator, CID-TLE—Complex Indicator of the Difficulty of the TLE.

## Data Availability

Readers can access the data supporting the conclusions of the study at www.usuwanieelektrod.pl (accessed on 25 October 2023).
